# Metabolomics Approach Revealed Polyunsaturated Fatty Acid Disorders as Pathogenesis for Chronic Pancreatitis−Induced Osteoporosis in Mice

**DOI:** 10.3390/metabo15030173

**Published:** 2025-03-03

**Authors:** Xinlin Liu, Fenglin Hu, Yunshu Zhang, Shurong Ma, Haihua Liu, Dong Shang, Peiyuan Yin

**Affiliations:** 1 Clinical Laboratory of Integrative Medicine, The First Affiliated Hospital of Dalian Medical University, Dalian 116000, China; 2 Institute (College) of Integrative Medicine, Dalian Medical University, Dalian 116044, China; 3 The First Affiliated Hospital, Zhejiang University School of Medicine, Hangzhou 310003, China; 4 Department of General Surgery, The First Affiliated Hospital of Dalian Medical University, Dalian 116000, China; 5 Guantao County People’s Hospital, Handan 057750, China

**Keywords:** chronic pancreatitis, osteoporosis, metabolomics, lipidomics

## Abstract

**Background:** Osteoporosis is frequently observed in patients with chronic pancreatitis, and both conditions are closely associated with systemic metabolic disorders. However, the underlying mechanisms linking chronic pancreatitis and osteoporosis remain unclear. **Methods:** In this study, we utilized high−performance liquid chromatography–mass spectrometry (HPLC−MS) to conduct metabolomics and lipidomics analyses on pancreatic, serum, and other tissues from a mouse model of chronic pancreatitis−induced osteoporosis (CP−OP), with the aim to elucidate the metabolism−related pathogenic mechanisms of CP−OP. **Results:** We identified over 405 metabolites and 445 lipids, and our findings revealed that several metabolites involving the tricarboxylic acid (TCA) cycle, as well as triacylglycerols and diacylglycerols with higher saturation, were significantly increased in the CP−OP model. In contrast, triglycerides with higher unsaturation were decreased. Differential pathways were enriched in n−3 long−chain polyunsaturated fatty acid metabolism in both pancreatic and bone tissues, and these pathways exhibited positive correlations with bone−related parameters. Furthermore, the modulation of these polyunsaturated fatty acids by Qingyi granules demonstrated significant therapeutic effects on CP−OP, as validated in mouse models. **Conclusions:** Through the metabolomics approach, we uncovered that disorders in polyunsaturated fatty acids play a critical role in the pathogenesis of CP−OP. This study not only enhances our understanding of the pathogenesis of CP−OP but also highlights the therapeutic potential of targeting polyunsaturated fatty acids as a future intervention strategy for osteoporosis treatment.

## 1. Introduction

Chronic pancreatitis (CP) is a fibroinflammatory syndrome that occurs due to a combination of genetic, environmental, and other risk factors. Its main pathological features include chronic inflammatory injury, interstitial fibrosis, the calcification of some tissues, and pancreatic duct dilatation [1]. When pancreatic tissue is damaged, secretory function is permanently impaired and systemic metabolism is significantly altered. Osteoporosis is a generalized bone disease with decreased bone density and altered bone microstructure, resulting in fractures [2]. The latest statistics show that around a quarter of patients with chronic pancreatitis suffer from osteoporosis (OP), while about two−thirds of patients with chronic pancreatitis have osteopenia or osteoporosis [3,4,5]. The ACG Clinical Guidelines: Chronic Pancreatitis and the ESPEN Clinical Nutrition Guidelines for Acute and Chronic Pancreatitis say that patients with chronic pancreatitis should be checked regularly for low bone density (osteoporosis) and given advice on how to prevent it [4,5]. Osteoporosis is a generalized bone disease with decreased bone density and altered bone microstructure, resulting in fractures [6,7].

Bone microstructural injuries are more insidious and are usually not detected until after a physical examination or a fragility fracture, especially hip and spine fractures, which can lead to disability [8]. The bone remodeling unit consists of a variety of cells, including osteoblasts and osteoclasts, which form a relative balance between the bone−forming effects of osteoblasts and the bone-resorbing effects of osteoclasts [8,9]. The etiology of osteoporosis is multifactorial, including insufficient vitamin D absorption, aging, and hormone secretion. The pathogenesis of chronic pancreatitis−induced osteoporosis (CP−OP) is intricate, involving systemic metabolic changes in bone microstructure remodeling and osteoporosis formation [9,10]. The bioenergetic metabolisms of osteoblasts and osteoclasts has garnered significant attention in recent years, as they are closely related to glucose, lipid, and amino acid metabolism. Alterations in lipidomics, a comprehensive study of lipids, have also been observed in osteoporosis, and supplementation with long−chain polyunsaturated fatty acids has been demonstrated to be beneficial for bone health [8,9,11,12,13,14,15,16].

We used metabolomics and lipidomics to investigate the metabolic changes in mice with chronic pancreatitis and the metabolic mechanism of pancreatic cleansing granules in treating chronic pancreatitis and alleviating CP−OP.

## 2. Materials and Methods

### 2.1. Reagents

Cerulein was purchased from Sigma−Aldrich (St. Louis, MO, USA). ELISA kits for TGF−β, BALP, TRACP−5b, and fecal elastase−1 were purchased from Shanghai Xitang Biotechnology Co., Ltd. (Shanghai, China). Total protein extraction kits and BCA protein concentration determination kits were purchased from Kaiji Biotech Co., Ltd. (Taizhou, China). The following antibodies were purchased from abcam (Cambridge, UK): a−SMA (ab787), collagen (ab260043), Fibronectin1 (ab5688), TGFβ1 (ab215715), TRAF3 (ab239357), Osteoprotegerin (ab203061), RANKL (ab62516), actin (ab8226), and secondary antibodies (ab6721). These antibodies were used at the following concentrations: sRANKL (0.25–0.5 μg/mL), Osteoprotegerin (1:300), TGF−β1 (1:1000), actin (1:5000), and other antibodies (1:2000). The following reagents for RT−qPCR analysis were all purchased from Aikang Rui Bioengineering Co., Ltd. (Changsha, China): RNA extraction reagent (AG21102), reverse transcription reagent (AG11711), and PCR reagent (AG11702). Qingyi granules were purchased from the First Affiliated Hospital of Dalian Medical University (Dalian, China). Mass spectrometry−grade acetonitrile, methanol, formic acid, isopropanol, and ammonium acetate were purchased from Fisher Scientific (Fair Lawn, NJ, USA). Mass spectrometry−grade sodium bicarbonate and methyl tert−butyl ether (MTBE) were purchased from Sigma−Aldrich (St. Louis, MO, USA). Ultrapure water (18.2 mΩ cm) was obtained using a Milli−Q purification system (Merck KGaA, Darmstadt, Germany).

### 2.2. Establishment of Animal Model

Male C57BL/6 mice aged 6–8 weeks with a bodyweight of around 20 g were used, and the care and use of animals were approved by the Animal Care and Use Committee of Dalian Medical University (AEE19001). The mice were randomly divided into a control group (*n* = 30) and a chronic pancreatitis model group (*n* = 30). The chronic pancreatitis model was induced by intraperitoneal injection of cerulein 50 μg/kg (Sigma−Aldrich, C9026) six times at one−hour intervals, three days a week. Control group mice were intraperitoneally injected with an equal number of times and volume of physiological saline. At weeks 4, 6, and 8, 10, mice from each of the control and chronic pancreatitis model groups were sacrificed three days after the last injection of cerulein or physiological saline. Subsequently, to investigate the therapeutic effect of Qingyi granules on chronic pancreatitis−induced osteoporosis, a second batch of mice was divided into a control group (*n* = 10), a chronic pancreatitis osteoporosis group (*n* = 10), and a Qingyi granules treatment group (*n* = 10). The chronic pancreatitis osteoporosis model was induced by the same method of intraperitoneal injection of cerulein 50 μg/kg (Sigma−Aldrich, C9026) six times at one−hour intervals, three days a week, for a total of 8 weeks. The Qingyi granules group was treated with Qingyi granules (1.3 g/kg) twice daily by oral gavage, starting from the second week of model establishment, while the control and chronic pancreatitis osteoporosis groups were given an equal volume of water by oral gavage. All groups of mice were sacrificed three days after the last injection of cerulein or physiological saline.

### 2.3. Sample Collection

Mice were anesthetized using isoflurane, and blood was collected via intraperitoneal venipuncture. Serum was obtained by centrifuging the blood at 1000 rpm for 5 min, with a portion used for ELISA detection and the remainder stored at −80 °C for metabolomic analysis. Mouse pancreatic tissue, femur, and feces were collected. The pancreatic tissue was divided into two parts: one part was fixed in paraformaldehyde for HE and Masson staining; the other part was quickly frozen in liquid nitrogen and stored at −80 °C for real−time fluorescent quantitative PCR, Western blot, and metabolomic analysis. The femur tissue was also divided into two parts: one part was fixed in paraformaldehyde for Micro−CT scanning, HE, and TRAP staining; the other part was quickly frozen in liquid nitrogen for Western blot and metabolomic analysis.

### 2.4. Sample Preparation for Metabolomics

150 μL of serum sample was transferred into a 1 mL 96−well deep−well plate, then 600 μL of methanol was added to precipitate the proteins. After vortexing and centrifugation, the upper 200 μL layers were transferred repeatedly into two 450 μL 96−well plates. For the preparation of pancreatic tissue/femur tissue samples, 20 mg of pancreatic tissue/femur tissue sample was weighed and 600 μL of 80% methanol was added. Then, 300 μL of the homogenate was transferred into a 2 mL printed tube and 900 μL of methyl tert−butyl ether (MTBE) and 150 μL of pure water were added, followed by vortexing, standing, and centrifugation, and drying using vacuum centrifugation. The polar metabolites in these two plates were redissolved with a polar solution for positive and negative ion detection and untargeted metabolomics analysis. Next, 20 μL of the sample was taken out and placed into a 1.5 mL EP tube with 120 μL of methanol. Then, the mixture was vortexed (1000 rpm for 5 min) and 360 μL of MTBE and 100 μL of ultrapure water were then added to the solution. After being stored at room temperature for 10 min and centrifuged, 200 μL of the supernatant was transferred into a 1.5 mL EP tube and dried. Finally, QC samples were prepared, and the extracts were redissolved for lipidomics analysis.

### 2.5. Untargeted Metabolomics and Lipidomics Analysis

We utilized the following UPLC−HRMS system: an Ultimate™ 3000 ultra−high−performance liquid chromatograph coupled with a Q Exactive™ quadrupole−orbitrap high−resolution mass spectrometer (Thermo Scientific, Waltham, MA, USA).

The UPLC separation conditions were as follows: Polar metabolite extracts were separated via reversed−phase chromatography for positive and negative ion detection. In the positive ion mode M1, the metabolites were separated using an AcquityTM HSS C18 column (Waters Co., Milford, MA, USA, 2.1 × 100 mm) with mobile phases A (0.1% formic acid in water) and B (0.1% formic acid in acetonitrile), with a gradient elution starting from 2% B and increasing to 98% within 10 min. In the negative ion modes M2 and M3, an AcquityTM BEH C18 column (Waters Co., USA, 1.7 μm, 2.1 × 100 mm) was used. For M2, the mobile phases were A (water/ammonium) and B (water/ammonium/acetonitrile/methanol), both containing ammonium bicarbonate buffer (concentration 0.4 g/L), starting from 2% B and increasing to 100% within 10 min. For M3, the mobile phases were A (5% water/acetonitrile) and B (40% acetonitrile/water). Ammonium acetate (concentration 0.8 g/L) was added as a buffer to improve separation, starting from 100% A and increasing to 10% within 12 min. The flow rate and column temperature were the same for M1 and M2: 0.4 mL/min, 50 °C; and for M3: 0.3 mL/min, 40 °C. Lipidomics metabolites were detected in the positive and negative ion detection modes, and the metabolites were separated using an Accucore C30 core–shell column with 60% acetonitrile aqueous solution (A) and 10% acetonitrile isopropanol solution (B) both containing ammonium formate and 0.1% formic acid, with a gradient elution starting from 10% B, increasing to 50% within 5 min, and further increasing to 100% within 23 min.

### 2.6. ELISA Analysis

According to the experimental procedures described in the ELISA kit, the levels of TGF−β, ALP, TRACP−5b, and fecal elastase−1 in mouse serum were determined.

### 2.7. HE Staining and Masson Staining

The following describes the staining procedures: First, remove the pancreatic tissue fixed in paraformaldehyde and dehydrate it through a gradient of ethanol. Then, embed and fix it using liquid paraffin, and section it with a paraffin microtome to a thickness of 4 μm. For HE staining, deparaffinize the samples in xylene and rehydrate them through a gradient of ethanol solutions before staining with hematoxylin and eosin. Dehydrate again through a gradient of ethanol, and mount with neutral resin to obtain the pancreatic tissue HE−stained sections. For Masson staining, soak the paraffin sections in ethylene glycol ether acetate and a gradient of ethanol to remove the embedding medium and rehydrate the embedding and resolubilizing agents. Stain with Masson staining reagents, wash, and mount with a neutral resin to obtain the pancreatic tissue Masson−stained sections. In Masson staining, collagen fibers appear blue, and the degree of pancreatic tissue fibrosis is assessed based on the area and extent of the blue−stained regions.

### 2.8. Micro−CT and Bone Histomorphometric Analysis

After fixing the mouse femur in 4% paraformaldehyde for 2 days, it was replaced with a 75% ethanol solution for soaking. Micro−CT scanning of the femur’s trabecular bone was performed using the Bruker Micro−CT Skyscan 1276 system (Kontich, Belgium). The scanner parameters were set as follows: resolution at 6 μm, X−ray tube voltage at 85 kV, and X−ray intensity at 200 μA. The femur was analyzed using three−dimensional model visualization software (CTvox; version 3.3.0), data analysis software (CT Analyser, version 1.18.8.0), and volume reconstruction software (Nrecon, version 1.7.4.2).

### 2.9. Western Blot Analysis

We prepared the samples via separation by 10% SDS−PAGE, followed by transferring them to a PVDF membrane and then blocking with 5% skim milk for 1 h. The PVDF membrane was incubated with various primary antibodies at 4 °C overnight, and then with secondary antibodies at room temperature for 1 h. The target proteins were visualized using an ECL system (Tanon, Shanghai, China), with β−actin serving as a control.

### 2.10. Real−Time Quantitative PCR Analysis

According to the instructions of the SYBR^®^Premix Ex Taq™II (TliRNaseH Plus) kit, we performed PCR reactions to detect the expression of Collagen1, a−SMA, and Fibronectin1 genes in pancreatic tissue, using β−actin as an internal control. Primer sequences were as follows: a−SMA—F: 5′−GCGTGGCTATTCCTTCGTGACTAC−3′; R, 5′−CGTCAGGCAGTTCGTAGCTCTTC−3′. Fibronectin1—F: 5′−AGTGGCTGAAGTCGCAAGGAAAC−3′; R: 5′−TAAGTCTGGGTCACGGCTGTCTC−3′. Collagen1—F: 5′−GACAGGCGAACAAGGTGACAGAG−3′; R: 5′−CAGGAGAACCAGGAGAACCAGGAG−3′. β−actin—F: 5′−CATCCGTAAAGACCTCTATGCCAAC−3′; F: 5′−ATGGAGCCACCGATCCACA−3′.

### 2.11. Metabolomics Data Processing

For polar small molecule metabolites, Compound Discoverer software was used to analyze metabolites, referencing the laboratory’s proprietary iPhenomeTM SMOL high−resolution MS/MS spectral library, the NIST 17 tandem MS/MS library (US National Institute of Standards and Technology), the mzCloud library (Thermo Scientific, USA), the Human Metabolome Database (HMDB), and the Kyoto Encyclopedia of Genes and Genomes (KEGG) for the structural annotation of polar small molecule metabolites. When identifying or annotating metabolites, mass accuracy was controlled within ±5 ppm, and in addition to exact mass, at least one isotope within 10 ppm and a fit score of relative isotope abundance of 70% were used to confirm the chemical formula. Furthermore, retention time information and high−resolution MS/MS spectral similarity were strictly used to confirm the structural annotation of the metabolites. On the other hand, LipidSearch software (Thermo Scientific™) was used to process the untargeted lipidomics data, including peak extraction and lipid compound identification. TraceFinder software (Thermo Scientific™) was used to extract the area under the curve (AUC) as relative quantitative information for the metabolites and lipids, with strict manual review and individual examination, mainly to eliminate false positives based on peak wobbling, adduct ion behavior, fragmentation patterns, and chromatographic behavior.

### 2.12. Statistical Analysis

When analyzing metabolomics data statistically, metabolites with missing values exceeding 50% of the sample size were removed. The remaining missing values were estimated and filled using the KNN sampling method. The measured dataset was then merged and trimmed for statistical analysis. The SPSS 21.0 software (SPSS, Inc., Chicago, IL, USA) and Student’s t−test were used to obtain *p*−values, which were corrected with the Benjamini–Hochberg false discovery rate (FDR) to obtain q−values (*p*−value < 0.05 and q−value < 0.05) for statistical analysis. Metaboanalyst was used for principal component analysis (PCA) and orthogonal partial least squares—discriminant analysis (OPLS−DA), and was employed to construct group−based models and identify metabolites enriched in inter−group differences. The obtained differential metabolites were uploaded to Metaboanalyst for the enrichment analysis of differential metabolic pathways. Heatmaps were created on Microbioinformatics, and Cytoscape 3.8.0 was used to display the network graph of n−3 polyunsaturated fatty acids and bone−related indicators.

## 3. Results

### 3.1. Establishment of Chronic Pancreatitis−Associated Osteoporosis Mouse Model

**Figure 1:**
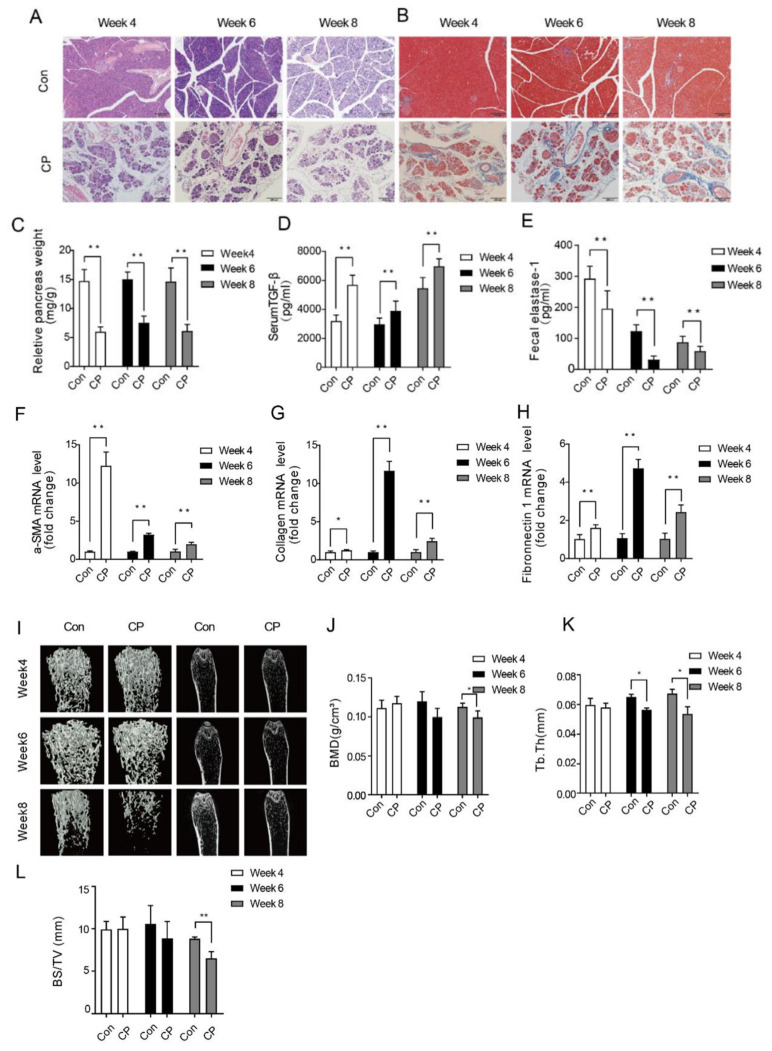
(**A**) HE staining of mouse pancreatic tissue at 4, 6, and 8 weeks. (**B**) HE and Masson staining results of mouse pancreatic tissue at the 4th, 6th and 8th weeks. (**C**) Relative weight of pancreas. (**D**) Concentration of TGF−β in serum. (**E**) Fecal elastase−1 content in feces. (**F**) a−SMA. (**G**) Collagen. (**H**) Fibronectin1. (**I**) Micro−CT of mouse femurs. (**J**) BMD analysis. (**K**) Tb.Th analysis. (**L**) BS/TV analysis. Data are presented as mean ± SD (*n* = 10 biologically independent samples; * *p* < 0.05, ** *p* < 0.01 by comparison with the control group.

Firstly, we established a chronic pancreatitis model by inducing it through daily intraperitoneal injections of cerulein 50 μg/kg at one−hour intervals, six times a day, for three days a week, over a period of 4 weeks. As shown in Figure 1A, with the extension of the modeling time, compared to the Con group, the relative weight of the pancreas in the CP group mice showed a decreasing trend. In the fourth week, HE staining of pancreatic tissue in the CP group showed the vacuolation of pancreatic acinar cells, destruction of normal pancreatic tissue, and a large accumulation of inflammatory cells around the tissue, with hemorrhage and necrosis in the pancreatic tissue (Figure 1A). The results of the Masson staining of pancreatic tissue are shown in Figure 1B, where the expression level of collagen fibers in the CP group’s pancreatic tissue is significantly increased. A large number of collagen fibers are distributed around pancreatic acinar cells and islet cells, with a significant amount of collagen fibers replacing the pancreatic parenchyma. Furthermore, the levels of TGF−β in mouse serum and fecal elastaCONse−1 in feces were detected, showing a significant increase in TGF−β levels (Figure 1D) and a decrease in fecal elastase−1 content (Figure 1E) in the CP group. Through the PCR analysis of the expression of fibrosis−related genes in pancreatic tissue, compared to the control group, the expression levels of the fibrosis−related genes a−SMA, collagen, and Fibronectin1 in the CP group mice’s pancreatic tissue were increased (Figure 1F–H). These results indicate that a chronic pancreatitis model was successfully induced by the method of intraperitoneal injection of cerulein by the fourth week. In order to study the pathogenesis of osteoporosis caused by chronic pancreatitis in mice, after the successful establishment of the chronic pancreatitis model, cerulein was continued to stimulate the mice, and the femur tissues of the mice were isolated at 4, 6, and 8 weeks, respectively, for Micro−CT examination to observe osteoporosis−related indicators. The Micro−CT of the mouse femurs showed that as the modeling time extended, the thickness and connectivity density of the trabecular bone gradually decreased (Figure 1I). As shown in Figure 1K, Tb.Th decreased in the 6th week. At the 8th week of the modeling, a significant decrease in BMD (Figure 1J) and BS/TV (Figure 1L) was observed in the CP group. These results indicate that a chronic pancreatitis osteoporosis model in mice was successfully established through 8 weeks of intraperitoneal cerulein injection.

### 3.2. Metabolic Characteristics of CP−OP Mice

**Figure 2:**
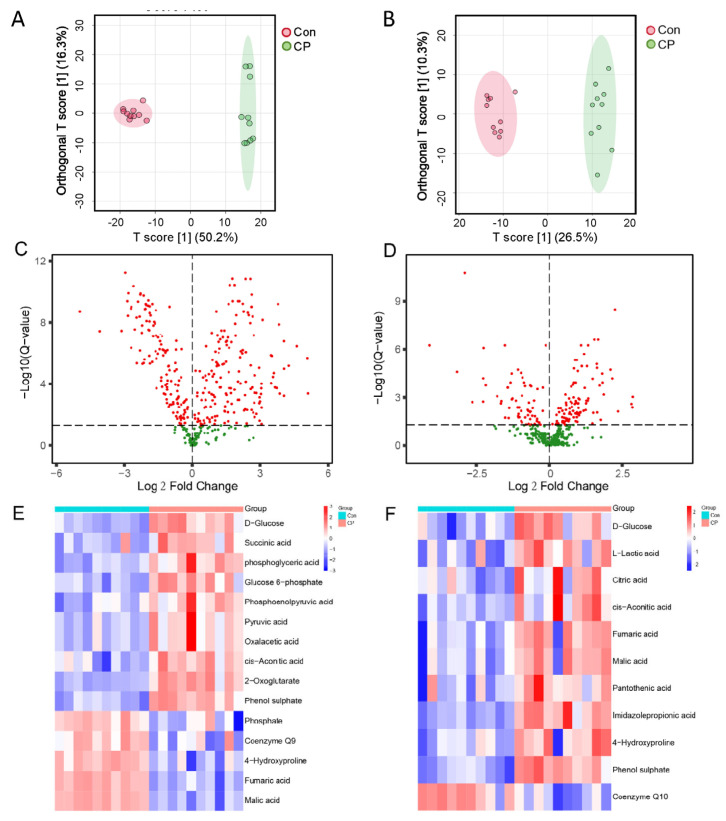
Polar small molecule metabolites in pancreatic tissue and serum of mice. (**A**) Pancreatic tissue OPLS−DA. (**B**) OPLS−DA of serum samples. (**C**) Volcanic map of pancreatic tissue. (**D**) Volcanic maps of serum samples. (**E**) Heat maps of metabolites associated with glucose metabolism in pancreatic tissue are presented. (**F**) Heat maps of metabolites associated with glucose metabolism in serum samples are presented.

Orthogonal partial least squares—discriminant analysis (orthoPLS−DA) based on multifactor analysis showed a clear separation and clustering trend in both the pancreatic and serum samples (Figure 2A,B), with the permutation verification results shown in Figure S1A,B [17]. Skeletal homeostasis is closely related to glucose metabolism [18]. In pancreatic tissue, metabolites related to glycolysis and the citric acid cycle, such as glucose, glucose−6−phosphate, glycerophosphate, phosphoenolpyruvate, pyruvate, oxaloacetate, citrate, α−ketoglutarate, and succinate, were all upregulated in the CP group, while fumarate and malate were downregulated (Figure 2E). In the serum, glucose, lactic acid, citric acid, cis−aconitic acid, fumaric acid, and malic acid in the CP group are all in an elevated state (Figure 2F). Urinary hydroxyproline, a product of collagen degradation, can serve as a marker of bone resorption [19,20]. Compared to the Con group, hydroxyproline in the pancreatic tissue of the CP group is decreased (Figure 2E), while hydroxyproline in the serum is increased (Figure 2F). Coenzyme Q9 and Coenzyme Q10 are important participants in the mitochondrial oxidative respiratory chain, providing energy for ATP synthesis and affecting the differentiation and function of osteoclasts and osteoblasts [21,22,23,24]. Coenzyme Q9 in pancreatic tissue and Coenzyme Q10 in serum were both downregulated in the CP group (Figure 2E,F). Metabolites related to diabetes, such as propionic acid and phenol sulfate, were all increased in the CP group (Figure 2E,F). Phosphate in the pancreas was significantly reduced in CP (Figure 2E), and pantothenic acid has a dual role in RANKL−induced osteoclast formation and development [25]. In the experiment, it was found that, compared to the Con group, the level of pantothenic acid in the serum of the CP group was upregulated (Figure 2F). Metabolic pathway analysis showed that differential metabolites were enriched in pathways such as the citric acid cycle; arginine biosynthesis; alanine, aspartate, and glutamate metabolism; and glycine, serine, and threonine metabolism.

### 3.3. Lipidomic Characteristics of CP−OP Mice

**Figure 3:**
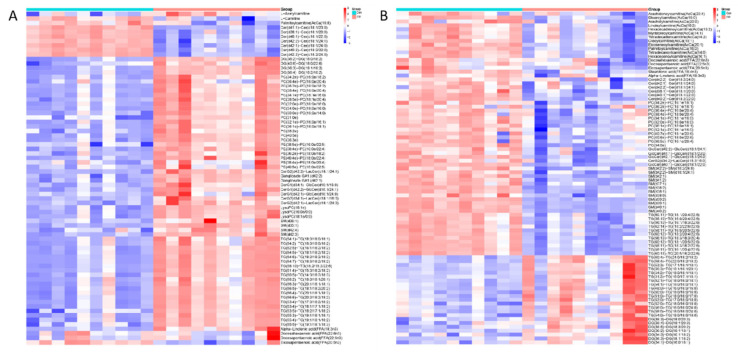
Lipid compounds in mouse pancreatic tissue and serum. (**A**) Heat map of lipid compounds in mouse pancreatic tissue. (**B**) Heat map of lipid compounds in mouse serum.

**Figure 4:**
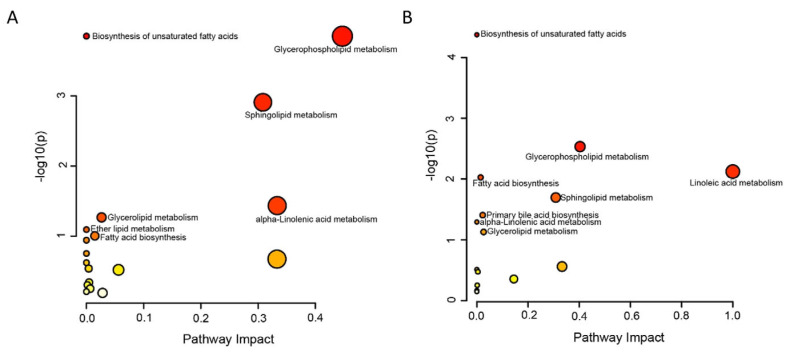
Metabolic pathways of lipid compounds with significant differences in mouse pancreatic tissue and serum were analyzed. (**A**) Bubble map of lipid pathway enrichment in mouse pancreatic tissue. (**B**) Bubble map of lipid pathway enrichment in mouse serum.

First of all, in mouse pancreas tissues, C2/C0 in the CP group increases compared with the Con group (Figure 3A), showing an increase in the rate of beta oxidation of fatty acids in pancreas tissue in a state of disease, but serum presents the opposite trend. C2/C0 in CP group is reduced and long−chain acyl carnitine content is lower (Figure 3B), because the serum represents the systemic metabolic state, suggesting that in chronic pancreatitis and osteoporosis, the mice are in an overall state of reduced fatty acid oxidation rate. Then, the activity of carnitine palmitoylcarnitine (CPT−1) was further considered. The ratio of free carnitine to palmitoylcarnitine and Stearoylcarnitine (C0/(C16 + C18) is the activity index of this enzyme [26]. C0/(C16 + C18) is upregulated in both the pancreatic tissue and serum of mice with the disease (Figure 3A,B), which indicates that the expression of carnitine palmitoyl transferase−1 is decreased in the disease state. As can be seen from Figure 3A, except for the decline of ceramides in the CP group, sphingomyelin, sphingolipid, diacylglycerol, triglyceride, ether phosphatidylcholine, ether phosphatidylcholine, and ether lysate phosphatidylcholine all increased. In serum, ceramides, sphingomyelin, sphingolipids, ether phosphatidylcholine and triglycerides with high unsaturation, such as TG (18:2/22:6/22:6) and TG (20:5/18:2/22:6), decreased in the disease group. However, triglycerides with high saturation, such as TG (18:0/18:0/18:1) and TG (18:0/18:1/18:1), and diacylglycerol were increased (Figure 3B). It has been suggested that TG (18:0/18:0/18:1) and TG (18:0/18:1/18:1) are closely related to osteoporosis [27]. Lipid metabolism pathways with significant differences in mouse pancreatic tissue and serum were enriched, respectively, and the results were shown in Figure 4A (pancreatic tissue), Figure 4B (serum), and Table 1.

**Figure 5:**
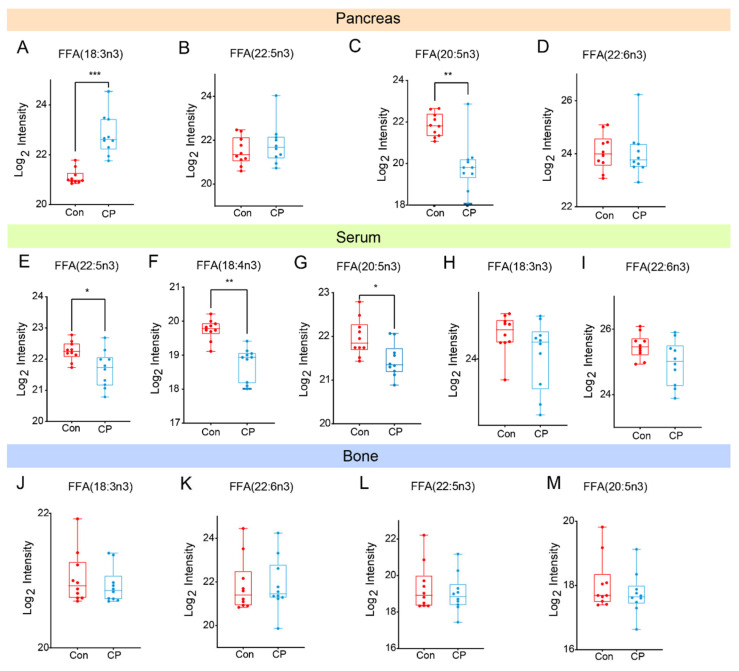

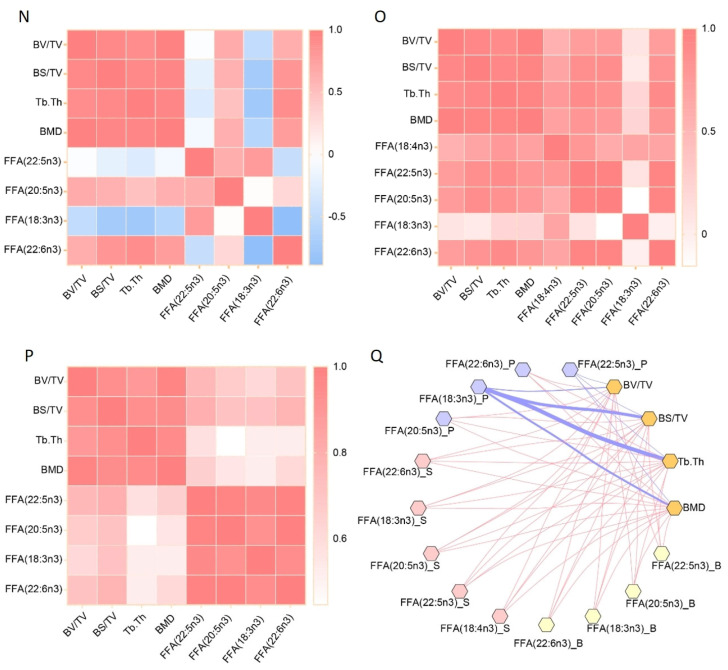
The relative content of n−3 fatty acids in pancreas, serum and bone. (**A**–**D**) The relative contents of n−3 fatty acids in the pancreas in each group. (**E**–**I**) The relative contents of n−3 fatty acids in serum in each group. (**J**–**M**) The relative contents of n−3 fatty acids in bone in each group. (**N**–**P**) The Pearson correlation chart of n−3 fatty acids and bone−related indexes in the pancreas, serum, and bone tissue. (**Q**) A correlation network diagram of n−3 fatty acids in the pancreas (_P), serum (_S), bone tissue (_B), and bone−related indexes. * *p* < 0.05, ** *p* < 0.01 by comparison with the control group.

Increasing dietary long−chain polyunsaturated fatty acids (especially n−3/omega−3 long−chain polyunsaturated fatty acids) contributes to bone health. This mechanism may be due to the promotion of osteoblast formation by downregulating PPARγ and enhancing osteoblast activity, and the inhibition of osteoclast formation by reducing the OPG/RANKL signaling pathway mediated by arachidonic acid−derived prostaglandin E2 [28,29,30]. The experimental results showed that the free n−3 long−chain unsaturated fatty acids in pancreatic tissues included α−linolenic acid (FFA (18:3n3), eicosapentaenoic acid (FFA (20:5n3)), docosahexaenoic acid (FFA (22:5n3)), and docosahexaenoic acid (FFA (22:6n3). FFA (18:3n3) increased significantly in the disease group (Figure 5A), and FFA (22:5n3) also showed an upward trend, but there was no statistical significance (Figure 5B). FFA (20:5n3) decreased significantly in the disease group (Figure 5C), and FFA (22:6n3) also showed a downward trend, but there was no statistical difference (Figure 5D). The free n−3 long−chain unsaturated fatty acids in serum include FFA (18:3n3), FFA (20:5n3), FFA (22:5n3), FFA (22:6n3), and linoleic acid FFA (18:4n3), of which FFA (22:5n3), FFA (18:4n3), FFA (18:4 N3), FFA (22:5 N3), FFA (18:4 N3), and FFA (20:5n3) decreased significantly in the CP group (Figure 5E–G), while FFA (18:3n3) and FFA (22:6n3) also showed a decreasing trend, but their differences were not statistically significant (Figure 5H,I). Among them, FFA (18:3n3) and FFA (22:5n3) were negatively correlated with bone−related parameters (bone volume fraction BV/TV, BS/TV, Tb.Th, BMD) (Figure 5N). Serum free n−3 long−chain unsaturated fatty acids, including FFA (18:3n3), FFA (20:5n3), FFA (22:5n3), FFA (22:6n3), and linoleic acid FFA (18:4n3), were positively correlated with bone parameters (Figure 5O). The n−3 long−chain polyunsaturated fatty acids in bone tissue were positively correlated with bone−related parameters (Figure 5O). The correlation between pancreas, serum, bone fat metabolism, and bone mass is shown in Figure 5Q.

### 3.4. Qingyi Granules Alleviate Lipid Metabolic Disorders Caused by CP−OP

**Figure 6:**
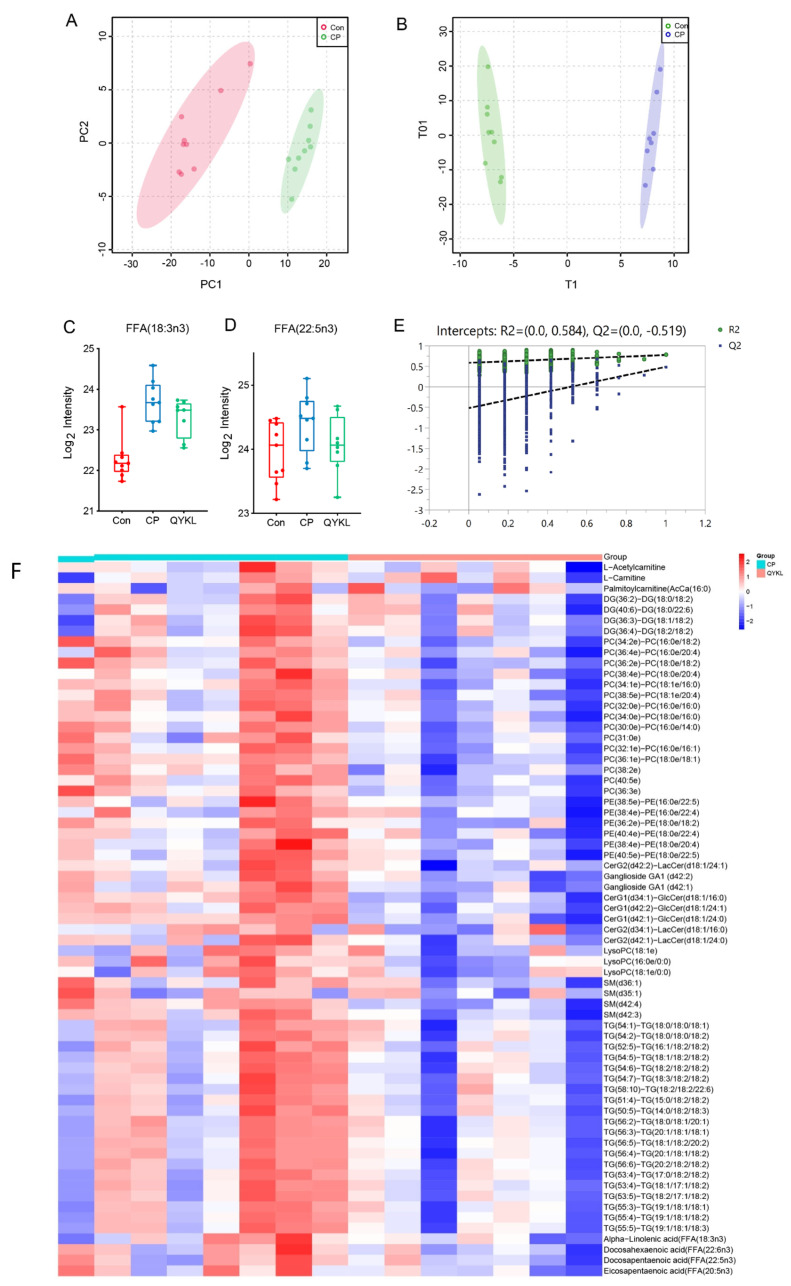
Qingyi granules improved lipid metabolism disorder of CP−OP. (**A**) PCA between Con group and CP group. (**B**) Orthogonal partial least squares—discriminant analysis of relative content of metabolites. (**C**,**D**) Relative content of α−linolenic acid (**C**) and docosapentaenoic acid (**D**) in serum. (**E**) Permutation verification of OPLS−DA model. (**F**) Heat map of relative content of metabolites between CP and QYKL groups.

The analysis results of lipidomics data are shown in Figure 6. PCA between the Con group and CP group showed an obvious separation trend between the two groups (Figure 6A). The separation effect of the PCA method between the CP group and QYKL group was poor, so the OPLS−DA method was used for analysis, and the results showed a good separation trend of OPLS−DA (Figure 6B). As mentioned above, FFA (18:3n3) and FFA (22:5n3) in mouse pancreatic tissue were negatively correlated with bone−related parameters (bone volume fraction BV/TV, BS/TV, Tb.Th, BMD) (Figure 5Q), that is, FFA (18:3n3) and FFA (22:5n3) increased in the disease group. Mice treated with Qingyi granules improved their accumulation of these two n−3 fatty acids in the disease state (Figure 6C,D), thus benefiting bone health. In addition, replacement tests (999 times) of the OPLS−DA model showed (Figure 6E) that the model had no overfitting phenomenon and had a high prediction accuracy. Similarly, ceramides decreased in the CP group, while sphingomyelin, sphingolipids, diacylglycerol, triglycerides, ether phosphatidylcholine, ether phosphatidylcholine, and lysophosphatidylcholine increased (Figure 3A), and in the QYKL group, these lipid compounds had varying degrees of correction (Figure 6F), thus improving lipid metabolism disorders.

### 3.5. Qingyi Granules Alleviate CP−OP in Mice

**Figure 7:**
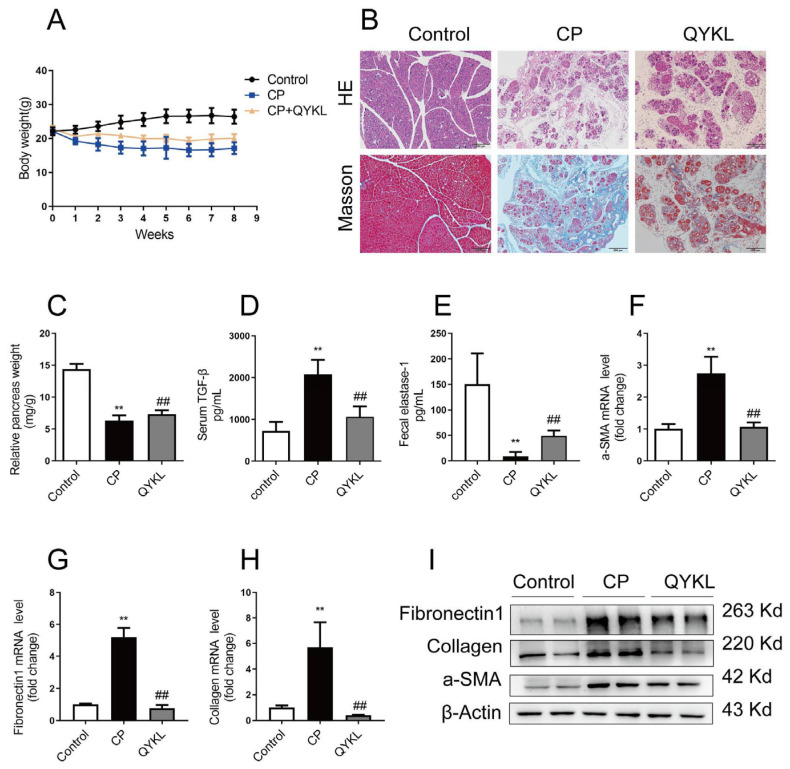
Qingyi granules attenuated CP and pancreatic fibrosis in mice. (**A**) The bodyweight changes in mice in the control, CP, and QYKL groups. (**B**) Representative images of HE and Masson staining of the pancreas. (**C**) The relative pancreas weights of the Con, CP, and QYKL groups. (**D**,**E**) The levels of TGF−β1 in the serum and fecal elastase−1 from the mice as determined by ELISA. Presented as mean ± SD (*n* = 8 biologically independent samples; * *p* < 0.05, ** *p* <0.01 by comparison with the control group and # *p* < 0.05, ## *p* < 0.01 by comparison with the CP group). (**F**–**H**) The qPCR analysis of αSMA, Collagen1, and Fibronectin1 in the pancreas. (**I**) The Western blot analysis of αSMA, Collagen1, and Fibronectin1 in the lysates of femoral metaphyses from the Con, CP, and QYKL groups.

**Figure 8:**
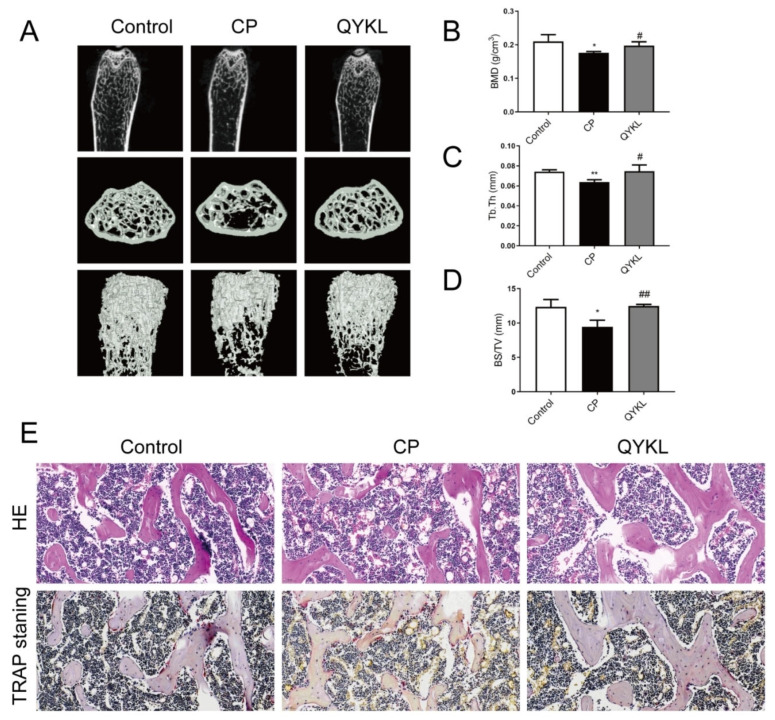
Qingyi granules relieved CP−induced osteoporosis. (**A**) Representative three−dimensional and two−dimensional pictures of trabecular bone in the distal femur. (**B**–**D**) The relative quantification of bone mineral density (BMD), trabecular thickness (Tb.Th), and trabecular bone surface density/total volume (BS/TV) in distal femurs from the control, CP, and QYKL mice. Presented as mean ± SD (*n* = 3 biologically independent samples; * *p* < 0.05, ** *p* <0.01 by comparison with the control group and # *p* < 0.05, ## *p* < 0.01 by comparison with the CP group). (**E**) Representative HE− and TRAP−stained images of femoral sections from the control, CP, and QYKL mice. The wine−red area after TRAP staining indicates osteoclasts.

Qingyi granules have been proven to have good therapeutic effects on acute pancreatitis and its complications in clinical trials and basic experimental studies [31,32]. Since about 60% of chronic pancreatitis cases evolve from acute and recurrent acute pancreatitis [5], weight loss in mice was slowed after treatment with Qingyi granules (Figure 7A). HE and Masson staining showed that after treatment with Qingyi granules the histopathological damage and fibrosis of the pancreas of mice were reduced compared with those in the CP group (Figure 7B). In addition, after treatment with Qingyi granules, the relative weight of the pancreas in mice increased compared with that in the CP group, the serum TGF−β decreased, and the content of fecal elastase−1 increased (Figure 7C–E). As shown in Figure 7F–I, Western blot and PCR analysis further confirmed that the protein and gene levels of a−SMA, Collagen1, and Fibronectin1 in CP−group mice were upregulated. The Micro−CT of bone showed that bone trabecular thickness and joint density gradually increased in the QYKL group compared with the CP group (Figure 8A). As shown in Figure 8B–D, BMD, Tb.Th, and BS/TV all show a reverse trend. The HE and TRAP staining of bone showed that Qingyi granules could increase the thickness of bone trabeculae and reduce the number of osteoclasts in bone tissue (Figure 8E).

## 4. Discussion

Fatty acids are produced from stored triglycerides, or fat pools, in response to lipolysis, when they are released into circulation and transported to the mitochondria for beta oxidative degradation through carnitine mediation to produce ATP [9]. Kim et al. explored the catabolism of fatty acids during bone formation by specifically disrupting the expression of carnitine palmitoyl transferase 2 (CPT2) in osteoblasts and osteocytes, and proved the necessity of fatty acid oxidation during bone accumulation and the role of bone in lipid homeostasis [33]. A study using fluorescently or radiolabeled chylomicron residues (CRs) showed that the capacity of bone to take up lipoprotein after a meal is second only to that of the liver, demonstrating that bone is involved in postprandibular lipoprotein metabolism in mice, and osteoblasts participate in circulating CR clearance, directly affecting the secretory function of osteoblasts [34]. In this study, n−3 polyunsaturated fatty acids could alleviate osteoporosis by inhibiting bone breakdown, promoting dietary calcium absorption, and reducing the production of prostaglandin E2 to promote healthy bone development [28,33,35]. In our previous work on a sodium taurinecholate−induced rat model, the biosynthesis of unsaturated fatty acids was upregulated and shown to be related to severe AP [36]. In this work, similar results are observed and restored by intervention with Qingyi granules, including emodin. n−3 polyunsaturated fatty acids in serum and bone are positively correlated with bone−related indicators. In the case of chronic pancreatitis and osteoporosis, BV/TV, BS/TV, Tb.Th, and BMD all decreased, and n−3 fatty acids all showed a decreasing trend. However, in pancreatic tissues, FFA (18:3n3) and FFA (22:5n3) were negatively correlated with the relevant indicators, and were upregulated in the disease group, suggesting that the excessive accumulation of FFA (18:3n3) and FFA (22:5n3) in the pancreas led to a sharp reduction in their levels in blood and bone, thus affecting the balance of bone formation and bone resorption. After using Qingyi granules, the lipid metabolism of mice was improved, and both pancreatitis and osteoporosis were alleviated. Studies have shown that in lipid metabolism disorders in osteoporosis patients, glycerophospholipids, sphingolipids, fatty acids and bile acids (BA) are mainly affected, and cholic acid (HCA), a key metabolite of lipid metabolism, has been found to play an important role in the occurrence of osteoporosis and may be a potential marker. These metabolites provide some basis for future research on the relationship between osteoporosis and fatty acid metabolism and bile acid metabolism [37].

Despite our efforts to elucidate the pathogenesis of CP−OP through metabolomics, this study still has some limitations. On the one hand, the identification of triglycerides is based on prediction. These limitations stem from the current constraints in lipid analysis methods and the lack of comprehensive lipid standards. Consequently, the identification of triglycerides primarily relies on existing databases and fragment prediction algorithms, which cannot distinguish between fatty acid chains at the 1, 3 and 2 positions. This issue remains unresolved within the scope of nontargeted metabolomics and is contingent upon advancements in analytical techniques. We have expanded our discussion on this matter in the revised manuscript as suggested. On the other hand, in our study, we found that the level of arachidonic acid was decreased during CP−OP. It has been reported that arachidonic acid and its related downstream products can activate inflammatory responses [38]. The abnormality of this phenomenon may be related to our modeling method, and further investigation into these mechanisms is needed in the future.

## 5. Conclusions

In this study, for the first time, a mouse model of chronic pancreatitis and osteoporosis was successfully established after 8 weeks of intraperitoneal injection of hyaline. Nontargeted metabolomics and lipidomics investigated the metabolic disorder caused by chronic pancreatitis and its association with the onset of osteoporosis, and Qingyi granules alleviated CO−OP by improving lipid metabolism. In summary, this study not only contributes to a better understanding of the pathogenesis of chronic pancreatitis osteoporosis, but also contributes to the development and clinical application of Qingyi granules.

## Figures and Tables

**Table 1:** Differential lipid pathway enrichment table in mice pancreases.

Class	Pathway Name	Match Status	*p*	−log(*p*)	FDR	Impact
Pancreas	Biosynthesis of unsaturated fatty acids	6/36	1.0 × 10^−4^	3.8522	6.00 × 10^−3^	0.45
	Glycerophospholipid metabolism	6/36	1.0 × 10^−4^	3.8522	6.00 × 10^−3^	0.45
	Sphingolipid metabolism	4/21	1.0 × 10^−3^	2.9089	3.00 × 10^−2^	0.31
	alpha−Linolenic acid metabolism	2/13	4.0 × 10^−2^	1.4349	8.00 × 10^−1^	0.33
	Glycerolipid metabolism	2/16	5.0 × 10^−2^	1.2673	9.00 × 10^−1^	0.03
	Ether lipid metabolism	2/20	8.0 × 10^−2^	1.0935	1.00 × 10^0^	0.01
	Fatty acid biosynthesis	3/47	1.0 × 10^−1^	1.0034	1.00 × 10^0^	0.01
Serum	Biosynthesis of unsaturated fatty acids	7/36	4.0 × 10^−5^	4.3749	4.00 × 10^−3^	0.4
	Glycerophospholipid metabolism	5/36	3.0 × 10^−3^	2.534	1.00 × 10^−1^	0.4
	Linoleic acid metabolism	2/5	8.0 × 10^−3^	2.1225	2.00 × 10^−1^	1
	Fatty acid biosynthesis	5/47	9.0 × 10^−3^	2.0259	2.00 × 10^−1^	0.01
	Sphingolipid metabolism	3/21	2.0 × 10^−2^	1.6957	3.00 × 10^−1^	0.31
	Primary bile acid biosynthesis	4/46	4.0 × 10^−2^	1.4049	6.00 × 10^−1^	0.02
	alpha−Linolenic acid metabolism	2/13	5.0 × 10^−2^	1.2933	6.00 × 10^−1^	0.02
	Glycerolipid metabolism	2/16	7.0 × 10^−2^	1.1296	8.00 × 10^−1^	0.03

## Data Availability

The data presented in this study are available in the main article; further inquiries can be directed to the corresponding author.

## Supplementary Materials

The following supporting information can be downloaded at: https://www.mdpi.com/article/10.3390/metabo15030173/s1, Figure S1: Permutation verification of OPLS-DA model for pancreatic tissue (A) and serum (B).

## Abbreviations

The following abbreviations are used in this manuscript:

CP−OP	Chronic pancreatitis−induced osteoporosis
PCA	Principal component analysis
CPT−1	Carnitine palmitoylcarnitine
PPARγ	Peroxisome proliferator−activated receptor gamma
QYKL	Qingyi granules
TGF−β	Transforming growth factor−beta
BALP	Bone alkaline phosphatase
TRACP−5b	Tartrate−resistant acid phosphatase 5b
TRAP	Tartrate−resistant acid phosphatase
ALP	Alkaline phosphatase
BV	Bone volume
TV	Tissue volume
Tb.Th	Trabecular thickness
Tb.N	Trabecular number
Tb.Sp	Trabecular separation/spacing
BMD	Bone mineral density
HE	Hematoxylin−eosin
a−SMA	Alpha−smooth muscle actin
