# Supplementary material for: Metabolomics Approach Revealed Polyunsaturated Fatty Acid Disorders as Pathogenesis for Chronic Pancreatitis−Induced Osteoporosis in Mice
Source: Metabolites. 2025 Mar 3;15(3):173. doi: 10.3390/metabo15030173 (PMC11944031; doi:10.3390/metabo15030173)
Supplement: Supplementary file 1 [file metabolites-15-00173-s001.zip › metabolites-3446108-supplementary.pdf]

Supplementary material

for

Metabolomics Approach Revealed Polyunsaturated Fatty Acids Disorders as Pathogenesis for Chronic Pancreatitis-induced Osteoporosis in Mice

Xinlin Liu [1,2], Fenglin Hu [2,3], Yunshu Zhang [1,2], Shurong Ma [1,4]*, Haihua Liu [1,5]*, Dong Shang [1,4] and Peiyuan Yin [1,4]*

Includes:
Figure S1A-B.

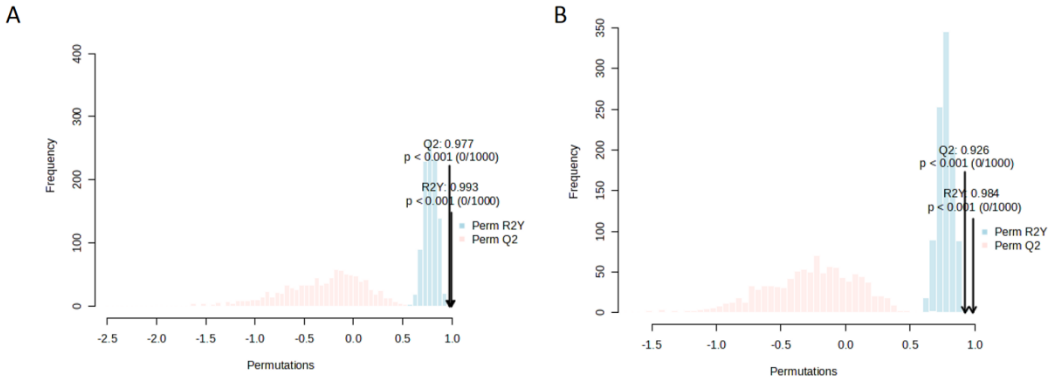

Figure S1. Permutation verification of OPLS-DA model for pancreatic tissue (A) and serum (B).
